# Degradation Factors and Mechanisms of Silicone Gel in Power Device Packaging Insulation Under DC Superimposed Pulse Electric Fields

**DOI:** 10.3390/gels12040274

**Published:** 2026-03-26

**Authors:** Zichen Wu, Dongxin He

**Affiliations:** Shandong Provincial Key Laboratory of UHV Transmission Technology and Equipment, School of Electrical Engineering, Shandong University, Jinan 250061, China; 202300190152@mail.sdu.edu.cn

**Keywords:** silicone gel, insulation degradation, DC superimposed pulse electric fields, electrical tree, space charge dynamic behavior

## Abstract

Silicone gel packaging for high-voltage power devices suffers severe insulation degradation under complex conditions involving sustained high voltages and steep pulses. DC superimposed pulse electric fields are the primary cause. However, existing research lacks a systematic quantitative analysis of key influencing factors. Motivated by this inadequacy, this study quantified the effects of four core factors via control variable-based electrical tree experiments and revealed the microscopic mechanism through charge vibration experiments. Results indicate that pulse voltage slew rate is the most critical factor, as the impact of altering the pulse voltage slew rate on the parameters of the electrical tree exceeds 200%, while the impacts of altering the superimposed DC amplitude and duty cycle are 49.92% and 77.56%, respectively. Further discussion demonstrates that pulse voltage slew rate reflects the charge dynamic behaviors, while DC amplitude and duty cycle reflect charge static accumulation, with charge dynamic behaviors posing a more significant effect. This work clarifies key control parameters for silicone gel insulation degradation and the intrinsic influence chain from influencing factors to molecular stress, charge dynamic behaviors, electrical tree growth and silicone gel insulation degradation, providing theoretical support and technical guidance for optimizing the design and enhancing the reliability of silicone gel in power electronic devices packaging insulation.

## 1. Introduction

With the advancement of high-voltage direct current transmission projects and the large-scale application of renewable energy generation and grid integration, high-voltage electronic technology is playing an increasingly vital role in power transmission and distribution networks [1,2]. High-voltage power electronic devices—with advantages such as high power density, compact size, and ease of operation—have been widely adopted in modern power systems such as DC transmission and energy internet [3,4]. Silicone gel combines excellent mechanical properties, chemical stability, and superior insulation performance, effectively meeting insulation encapsulation requirements such as dustproofing, shockproofing, and moisture resistance [5,6]. It is well-suited as an insulation encapsulation material for power devices and has gained extremely widespread application in various electronic components [7,8,9,10].

Power devices in high-voltage direct current transmission systems operate under complex conditions involving sustained high voltages and steep pulses and must withstand not only the rated DC voltage but also pulse voltage during operation [11,12]. Under the influence of DC superimposed pulse voltage, the service life of silicone gel insulating materials falls far below their expected lifespan, accelerating insulation degradation [13,14]. The prominent characteristics of DC superimposed pulse voltage are its high amplitude and the instantaneous voltage surge at the edges [15]. In modern power electronic systems, especially with the widespread adoption of wide-bandgap semiconductor devices, insulation materials are increasingly subjected to fast transient voltages with higher frequencies and steeper slopes [16,17]. These transient electrical stresses have been recognized as a critical factor accelerating insulation degradation and introducing new reliability challenges [18,19,20]. At the microscopic level, recent studies have shown that under AC–DC composite or pulsed electric fields, charge dynamic behaviors—including charge injection, accumulation, and relaxation—can induce significant local electric field distortion, thereby promoting streamer discharge and electrical tree propagation in insulating materials [21]. This undoubtedly provides insights for our analysis of the microscopic mechanisms of the macroscopic insulation degradation phenomenon. From an engineering perspective, the reliability of power electronic device packaging is governed by multi-physical stress coupling, including electrical, thermal, chemical, and mechanical stresses [22,23]. So, stress quantification and condition monitoring should be highlighted in understanding insulation degradation under these complex conditions.

Previous experiments have already conducted preliminary investigations into the factors influencing insulation degradation. Zhang Xiaoqin et al. investigated factors affecting the degradation performance of oil–paper insulation, demonstrating that partial discharge voltage and discharge duration impact insulation degradation [24]. Chen Jianfu et al. investigated the factors influencing insulation degradation in power electronic transformers, demonstrating that aging time is one of the factors affecting insulation degradation [25]. Sun Hao et al. investigated the factors influencing the degradation characteristics of epoxy resin insulation in dry-type valve-side bushings of converter transformers, demonstrating the effects of harmonics and temperature [26]. He Dongxin, Zhang Tao et al. investigated the vibration patterns of space charge under different pulse rise times, demonstrating that the pulse rise time affects insulation degradation [27]. Xu Zhe investigated the electrical branching degradation characteristics of silicone elastomer under thermally coupled pulse electric fields, finding that the pulse voltage repetition frequency, pulse edge time, voltage amplitude, and temperature all influence the material’s insulation degradation [28]. Wei Junyu investigated the experimental patterns of square-wave pulse edge charges exciting molecular vibrations, discovering that the edge duration, maximum edge slope, and amplitude of the square-wave pulse electric field influence insulation breakdown [11]. The aforementioned studies have identified multiple key factors driving the insulation degradation of materials such as silicone gel in power devices, including partial discharge parameters, aging time, harmonics, temperature, and pulse voltage characteristics (rise time, amplitude, frequency, duty cycle), and preliminarily revealed their influence mechanisms. However, these studies have only examined a single characteristic of DC or pulse voltage. That is, they have investigated the impact of a single influencing factor on material insulation degradation without conducting a detailed, systematic analysis of all factors. Furthermore, they have failed to quantify and compare the relative significance of different factors on material insulation degradation. Consequently, the most critical factor affecting silicone gel insulation degradation remains unclear. The main reasons for this research gap lie in the fact that the simultaneous and precise regulation of multiple influencing factors demands high integration and accuracy from the experimental platform. Additionally, dynamic microscopic processes cannot be observed in real time, and the high dispersion of test data makes it difficult to extract reliable patterns. Furthermore, existing analyses of the impact of pulse rise time struggle to achieve nanosecond-level precision, thus failing to adequately address more severe operating conditions and providing practical guidance.

Therefore, we identified four critical factors: pulse voltage variation, superimposed DC amplitude, rise time (accurate to the nanosecond level), and duty cycle. We also established an electrical tree test platform and a charge vibration test platform. For each influencing factor, the variable control method was employed to determine the magnitude of its individual impact. Through quantification and comparison, we finally identified pulse voltage slew rate as the most critical factor, clarified the relationship between these influencing factors and charge dynamic behaviors, and revealed the failure mechanisms of silicone gel materials under DC superimposed pulse electric fields. This approach aimed to provide new strategies for mitigating insulation degradation in silicone gel materials and offer more precise guidance for the molecular design of insulating materials.

## 2. Results and Discussion

### 2.1. Electrical Tree Experiment

#### 2.1.1. At the Same Pulse Rise Time, the Pulse Variation Value Has the Greatest Impact, Followed by the Superimposed DC Amplitude, and Finally the Duty Cycle

**Figure 1:**
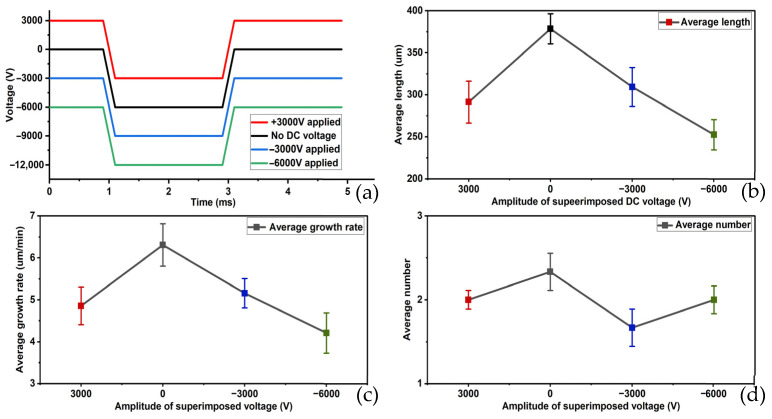
Patterns of changes in average length, average growth rate, and average initiation number of electrical tree at different superimposed DC amplitudes. (**a**) Schematic diagram of different superimposed DC amplitudes, (**b**) change in average length, (**c**) change in average growth rate and (**d**) change in average initiation number.

The results of the first set of experiments are shown in Figure 1. Under pulse voltages ranging from 3000 V to −3000 V: The average length of the electrical tree was 291.262 μm, the average growth rate was 4.854 μm/min, the average initiation number was two, and the average initiation time occurred instantaneously upon pulse voltage application. For pulse voltages ranging from 0 to −6000 V: the average length was 378.378 μm, the average growth rate was 6.306 μm/min, the average initiation number was two, and the average initiation time occurred at the instant the pulse voltage was applied. From −3000 V to −9000 V pulse voltage: the average length was 309.278 μm, the average growth rate was 5.155 μm/min, the average initiation number was two, and the average initiation time occurred at the instant of pulse voltage application; from −6000 V to −12,000 V pulse voltage: the average length was 252.427 μm, the average growth rate was 4.207 μm/min, the average initiation number was two, and the average initiation time occurred at the instant of pulse.

**Figure 2:**
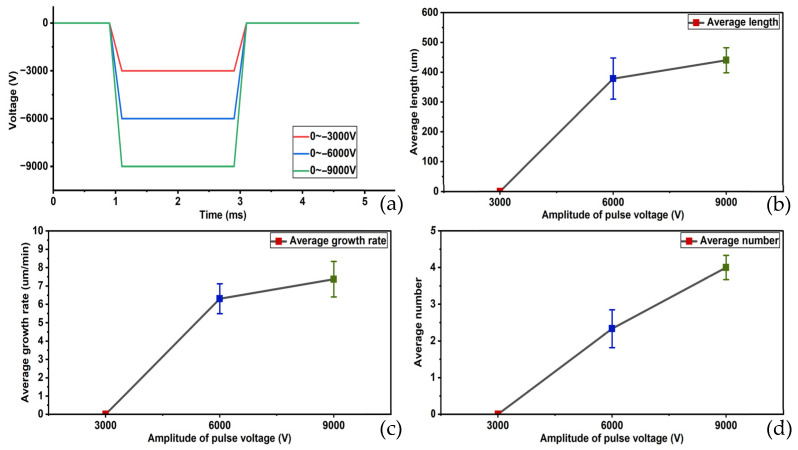
Patterns of changes in average length, average growth rate, and average initiation number of electrical tree at different pulse variation values. (**a**) Schematic diagram of different pulse variation values, (**b**) change in average length, (**c**) change in average growth rate and (**d**) change in average initiation number.

The results of the second set of experiments are shown in Figure 2. Under a pulse voltage of 0 to −3000 V, no tree occurred. At a pulse voltage of 0 to −9000 V, tree occurred at the instant the pulse voltage was applied, followed by rapid breakdown. The average length of electrical tree was 442.105 um, with an average growth rate of 7.368 μm/min. The average initiation number was four, and the average initiation time was the instant the pulse voltage was applied.

**Figure 3:**
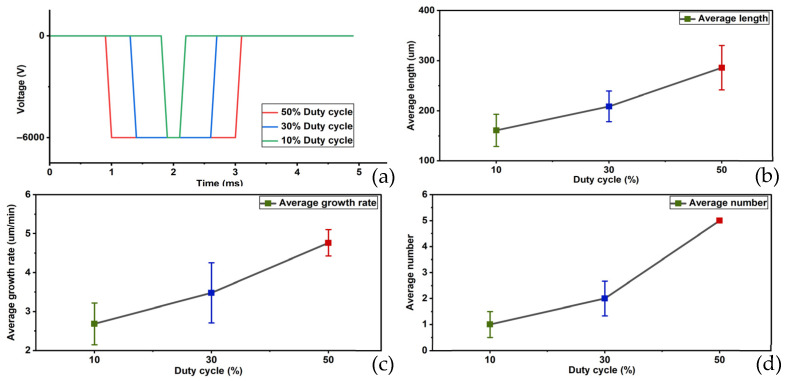
Patterns of changes in average length, average growth rate, and average initiation number of electrical tree at different duty cycles. (**a**) Schematic diagram of different duty cycles, (**b**) change in average length, (**c**) change in average growth rate and (**d**) change in average initiation number.

The results of the third group of experiments are shown in Figure 3: At a duty cycle of 50%, the average length of electrical tree was 285.714 μm, the average growth rate was 4.762 μm/min, the average initiation number was 5, and the average initiation time was the instant of pressure application. The growth rate of the electrical tree was relatively fast, and the formation of the electrical tree was more pronounced. At a 30% duty cycle, the average length of electrical tree was 208.696 μm, with an average growth rate of 3.478 μm/min, an average initiation number of two, and an average initiation time occurring at the instant of voltage application. The growth rate was slower than in the previous group, and the formed branches were smaller. At a duty cycle of 10%, the average length of the electrical tree was 160.920 μm, with an average growth rate of 2.682 μm/min. The average initiation number was one, and the average initiation time was 5.5 min. The growth rate of electrical tree was further reduced, and the formed branches were very small. No breakdown phenomena occurred in any of the experiments in the third group.

Therefore, by keeping other parameters constant and varying only the pulse variation value, superimposed DC amplitude, and duty cycle, differences in the electrical tree behavior of the silicone gel sample were observed. This indicates that all three factors influence the insulation degradation of the silicone gel sample, albeit to varying degrees. The overall influence pattern reveals that as the pulse variation value, the superimposed DC amplitude and the duty cycle increase, the treeing phenomenon on the sample becomes more pronounced, and the insulation degradation of the silicone gel sample becomes more evident.

This experiment aims to demonstrate the magnitude of the impact of three influencing factors—pulse variation, superimposed DC amplitude, and duty cycle—on the degradation of silicone gel insulation. However, when altering each factor individually, the corresponding three sets of data exhibit significant numerical differences. Plotting these on a linear coordinate axis makes it difficult to visually assess their relative effects and increases susceptibility to interference from individual outliers. Therefore, the y-axis scale employs a common logarithm to mitigate these issues.

**Figure 4:**
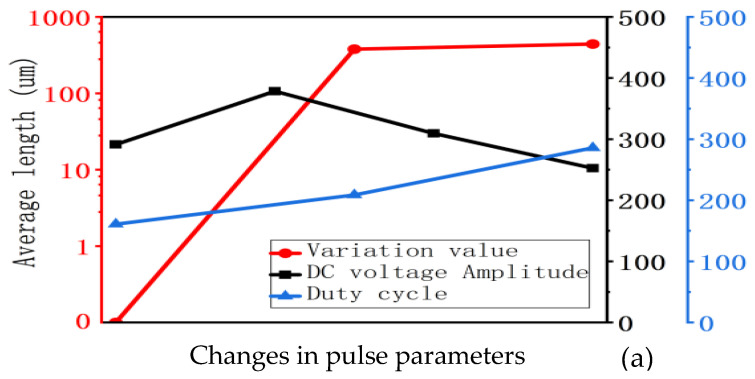

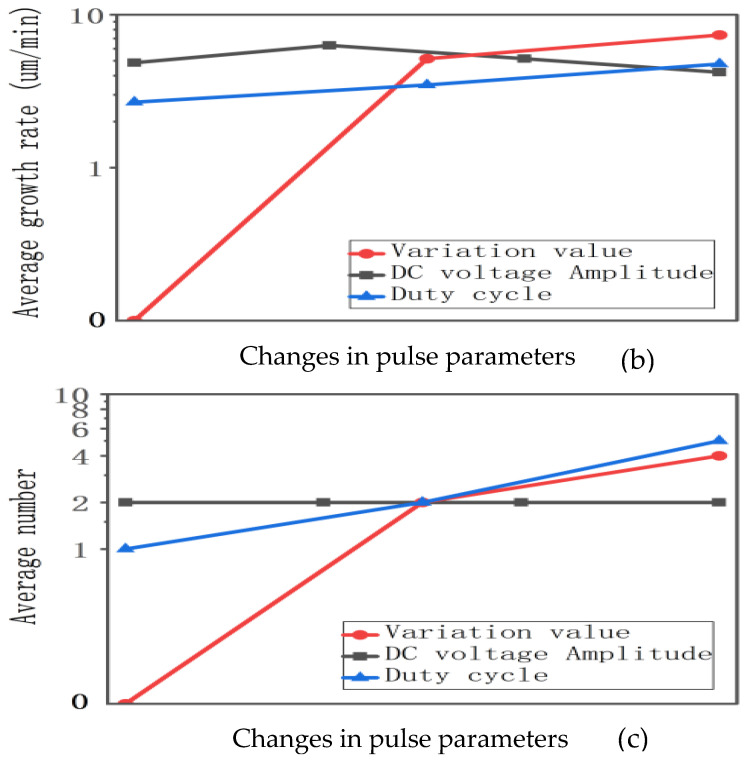
Comparison of the magnitude of the impact of pulse variation value, superimposed DC amplitude, and duty cycle. (**a**) Impact on average length, (**b**) impact on average growth rate and (**c**) impact on average initiation number.

For the three quantitative parameters: length, growth rate, and initiation number of electrical trees. The effects of altering three influencing factors on a single quantitative parameter are compared in a single graph. Figure 4 illustrates the changes in the length, growth rate, and initiation number of electrical trees when varying the pulse variation value, superimposed DC amplitude, and duty cycle, respectively. This allows for a direct comparison of the magnitude of the impact of these three factors.

As can be clearly seen from the figure above, when the rising edge time remains constant, the impact of pulse variation on the insulation degradation of silicone gel samples is greater than that of superimposed DC amplitude, and the impact of superimposed DC amplitude is greater than that of duty cycle.

#### 2.1.2. The Rise Time of the Pulse Has a Greater Impact than the Pulse Variation Value, Making the Pulse Voltage Slew Rate the Most Critical Influencing Factor

The aforementioned experiments have demonstrated that for silicone gel samples, the impact of pulse variation magnitude on insulation degradation is greater than that of superimposed DC amplitude. Since the pulse voltage variation rate comprises two components—pulse variation magnitude and rise time—this experiment will focus on and quantify the influence of rise time on insulation degradation. This will enable a comprehensive assessment of the overall impact of pulse voltage variation rate on insulation degradation.

**Figure 5:**
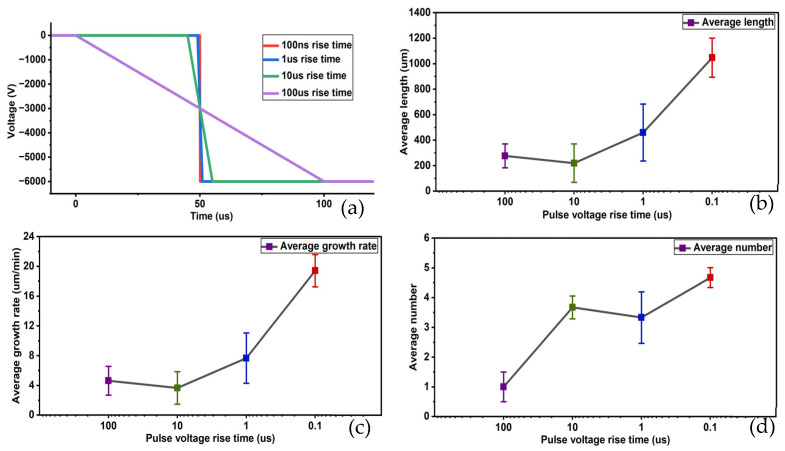
Patterns of changes in average length, average growth rate, and average initiation number of electrical tree at different rising edge times. (**a**) Schematic diagram of different rising edge times, (**b**) change in average length, (**c**) change in average growth rate and (**d**) change in average initiation number.

The experimental results are shown in Figure 5.

When the rising edge time is 100 ns, which corresponds to a slope of $-6\times 10^{7}\;kV/s$, the average length of electrical tree is 1046.875 μm, with an average growth rate of 19.412 μm/min. The average number of electrical tree initiated is 4.67, and the average initiation time is at the instant of voltage application. The average number of electrical trees that are broken down is one, with an average breakdown time of 10.83 min. The electrical tree grows rapidly, forming distinctly visible structures, and breakdown phenomena occur.

When the rising edge time is 1 μs, corresponding to a slope of $-6\times 10^{6}\;kV/s$, the average length of electrical tree is 459.770 μm, the average growth rate is 7.663 μm/min, the average number of electrical tree initiated is 3.33, and the average initiation time is 3 min. No breakdown phenomena occurred. Electrical tree growth primarily occurred within the first 30 min, with growth nearly ceasing in the subsequent 30 min. Compared to the previous group, the growth rate of electrical tree decreased, and the size of branches diminished.

When the rising edge time is 10 μs, corresponding to a slope of $-6\times 10^{5}\;kV/s$, the average length of electrical tree is 219.178 μm, the average growth rate is 3.653 μm/min, the average number of electrical tree initiated is 3.67, and the average initiation time is 1 min. No breakdown phenomena occurred. Electrical tree branch growth primarily occurred within the first 10 min, with growth nearly ceasing during the subsequent 50 min. The growth rate further decreased, and the resulting branches were smaller in size.

When the rising edge time is 100 μs, corresponding to a slope of $-6\times 10^{4}\;kV/s$, the average length of the electrical tree is 276.923 μm, the average growth rate is 4.615 μm/min, the average number of electrical tree initiated is one, and the average initiation time is 13.3 min. No breakdown phenomena occurred. The growth rate of the electrical tree is extremely slow, resulting in very small branches.

In summary, while keeping other parameters constant and varying only the rise time, the four experimental groups exhibited significant differences in electrical tree length, growth rate, number of electrical tree initiated, initiation time, number of electrical tree broken down, and breakdown time. This indicates that rise time is a key factor influencing sample insulation degradation [29]. Compared to other influencing factors, the impact of rise time on sample insulation degradation is particularly pronounced. The overall influence pattern shows that as the rise time decreases, the electrical tree branches on the samples become more pronounced, and the insulation degradation of the silicone gel samples becomes more evident.

**Figure 6:**
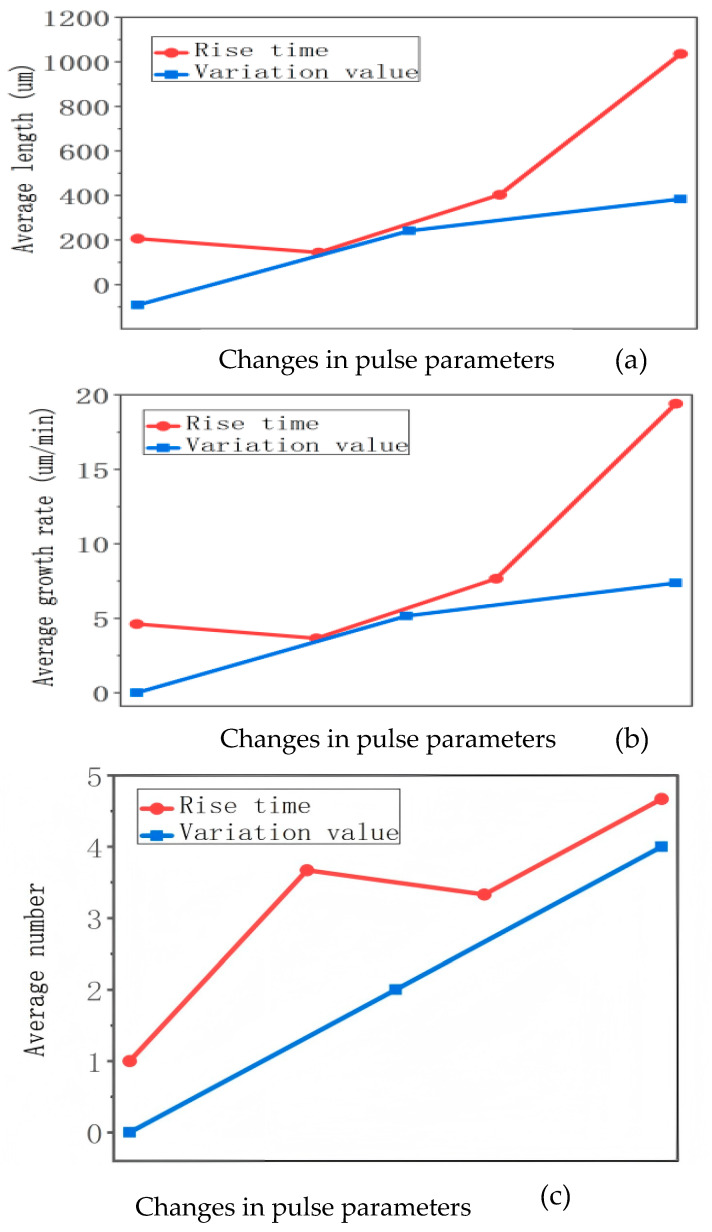
Comparison of the magnitude of the impact of between rising edge time and pulse variation value. (**a**) Impact on average length, (**b**) impact on average growth rate and (**c**) impact on average initiation number.

This experiment aims to demonstrate the magnitude of the impact of two influencing factors—pulse rise time and pulse variation value—on the degradation of silicone gel insulation. For three quantitative parameters: electrical tree length, growth rate, and initiation number, the effects of altering rise time and pulse variation value on each parameter are compared within a single graph. Figure 6 illustrates the changes in average length, average growth rate, and average number of tree roots formed when altering the rise time and pulse variation value separately. This allows for a direct comparison of the magnitude of the influence of these two factors.

As can be clearly seen from the figure above, under conditions where other factors remain constant, the rise time of the pulse has a greater impact on the insulation degradation of silicone gel samples than the pulse variation value.

**Figure 7:**
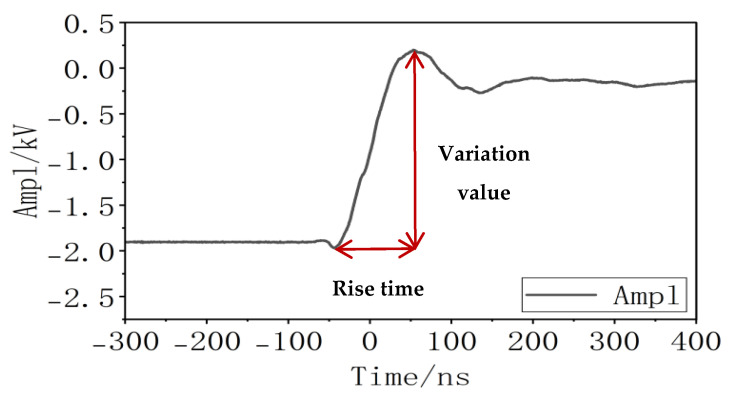
Pulse voltage rising edge diagram.

As shown in Figure 7, the pulse voltage slew rate comprises two aspects—the pulse change value and the rise time. As demonstrated in Section 2.1.1 of this paper: when the rise time remains constant, the influence of the pulse change value is greater than that of the superimposed DC amplitude, and the influence of the superimposed DC amplitude is greater than that of the duty cycle. The conclusion drawn from this experiment is that the rise time of the pulse has a greater impact than the pulse change value. Therefore, relative to other influencing factors, both the pulse change value and the rise time exert the most significant effects on the insulation degradation of the silicone gel samples. Ultimately, the pulse voltage slew rate, composed of both the pulse variation value and the rise time, is the most critical influencing factor.

To evaluate the effects of pulse voltage variation, superimposed DC amplitude, rise time, and duty cycle on the growth characteristics of electrical tree in silicone gel packaging insulation, statistical significance tests were conducted. An analysis of variance was employed with a significance level of *p* < 0.05. The results indicated that pulse voltage variation, superimposed DC amplitude, rise time and duty cycle showed a significant influence on three pulse parameters (*p* < 0.05).

### 2.2. Charge Vibration Experiment

The electrical tree experiment has demonstrated that for silicone gel insulation degradation, the rate of change in pulse voltage exerts the most significant influence, while the effects of DC amplitude and duty cycle are relatively minor. However, the electrical tree experiment focuses on comparing the magnitude of effects at the macroscopic level for studying insulation degradation phenomena, lacking investigation into the microscopic mechanisms of degradation. Therefore, to elucidate the complete chain from influencing factors to insulation degradation, thereby further validating the aforementioned experimental conclusions and gaining a deeper understanding of the degradation mechanism, a charge vibration experiment is conducted below.

#### 2.2.1. The Impact of Pulse Voltage Slew Rate (Pulse Voltage Slope) Is Greater than That of Superimposed DC Amplitude, and the Impact of Superimposed DC Amplitude Is Greater than That of Duty Cycle

**Figure 8:**
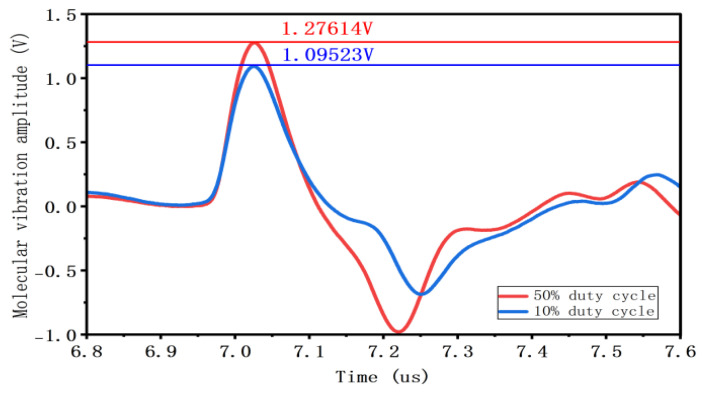
Charge vibration waveform and amplitude at different duty cycles.

As shown in Figure 8, when the superimposed DC amplitude remains constant, the amplitude of charge vibration increases as the pulse voltage duty cycle rises. Without superimposed DC voltage, as the duty cycle increases from 10% to 30% and then to 50%, the charge vibration amplitude increased from 1.09523 V to 1.25815 V and 1.27614 V. When a DC voltage was superimposed, increasing the duty cycle from 10% to 30% and 50% resulted in the charge vibration amplitude increasing from 3.43462 V to 3.43937 V and 3.47260 V.

**Figure 9:**
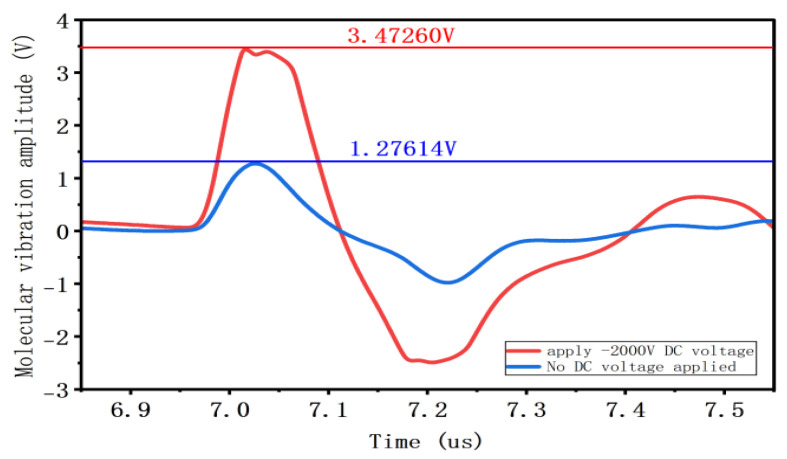
Charge vibration waveform and amplitude at different superimposed DC amplitudes.

As shown in Figure 9, when the duty cycle remains constant, the amplitude of charge vibration increases with the rise in superimposed DC voltage amplitude. At a 10% duty cycle, applying a −2000 V DC voltage increases the charge vibration amplitude from 1.09523 V to 3.43462 V. At a duty cycle of 30%, superimposing a −2000 V DC voltage increases the charge vibration amplitude from 1.25815 V to 3.43937 V; at a duty cycle of 50%, superimposing a −2000 V DC voltage increases the charge vibration amplitude from 1.27614 V to 3.47260 V.

**Figure 10:**
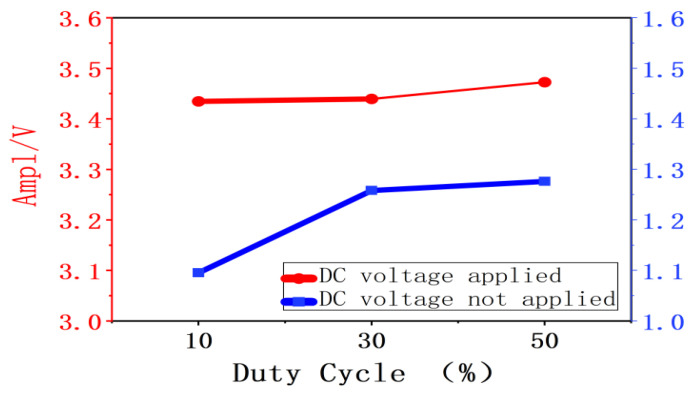
Patterns of variations in charge vibration amplitude at different duty cycles and superimposed DC amplitudes.

As shown in Figure 10, the amplitude of charge vibration increases both with rising duty cycle and with increasing superimposed DC amplitude. This clearly demonstrates that both the superimposed DC amplitude and the duty cycle influence the amplitude of charge vibration.

**Figure 11:**
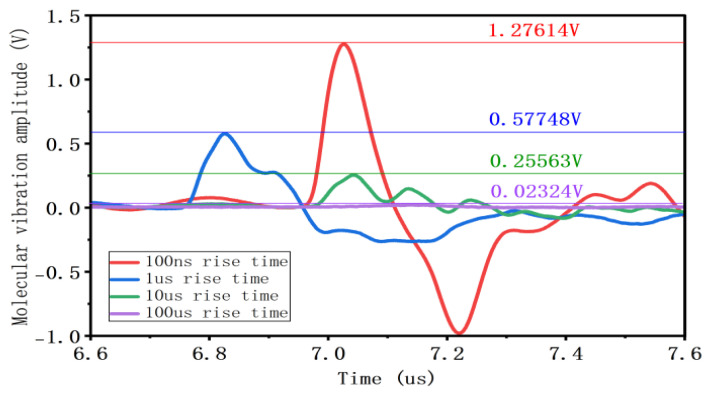
Charge vibration waveform and amplitude at different rising edge times.

As shown in Figure 11, increasing the pulse rise slope results in a larger vibration amplitude. As the rise time increases from 100 ns to 1 μs, 10 μs, and 100 μs, corresponding to pulse rise slope rates of $-6\times 10^{7}\;kV/s$, $-6\times 10^{6}\;kV/s$, $-6\times 10^{5}\;kV/s$ and $-6\times 10^{4}\;kV/s$, the amplitude of the charge vibration significantly increases from 0.02324 V to 0.25563 V, 0.57748 V, and 1.27614 V, respectively.

**Figure 12:**
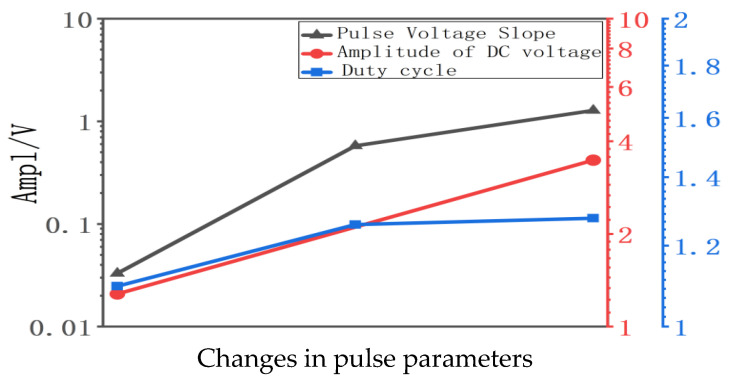
Comparison of the influence magnitude of pulse slew rate, superimposed DC amplitude, and duty cycle.

Below, we compare the quantified impact of three influencing factors—pulse slew rate, superimposed DC amplitude, and duty cycle—on the vibration amplitude parameter in a single diagram. Figure 12 visually compares the quantified impact of three influencing factors on the vibration amplitude of charge. This figure clearly demonstrates that the pulse slew rate exerts a greater influence than both the superimposed DC amplitude and the duty cycle.

To evaluate the effects of pulse slew rate, superimposed DC amplitude, and duty cycle on the charge vibration amplitude in silicone gel packaging insulation, statistical significance tests were conducted. An analysis of variance was employed with a significance level of *p* < 0.05. The results indicated that pulse slew rate, superimposed DC amplitude and duty cycle had significant effects on the charge vibration amplitude (*p* < 0.05).

#### 2.2.2. Theoretical Analysis of Forces Acting on Charges

Charges in a pulse electric field are subjected to both electric forces and material stresses. The electric field force is the force exerted on a charge within the electric field, while material stress encompasses the combined forces between charges, including Coulombic forces, molecular chain confinement forces, and the attraction of atomic nuclei on electrons. The vibration of charges occurs under the combined force of these two components. Therefore, the mechanism of charge vibration during the rising and falling edges can be elucidated by analyzing the resultant force acting on the charges.

Changes in resultant force can be roughly calculated using the work–energy theorem [26]. The formula for the work–energy theorem is as follows:

$$F\cdot \Delta t=\mathrm{mv}-{\mathrm{mv}}_{0}$$

Here, F represents the net electric force acting on the charge, Δt denotes the time interval, m is the charge’s mass, v is the charge’s velocity at the final state, and v_0_ is the charge’s velocity at the initial state. Charges moving at higher velocities generate stronger ultrasonic waves, resulting in higher voltages measured by the PVDF sensor. Therefore, the voltage amplitude detected by the PVDF sensor directly reflects the charge’s velocity.

During the constant-voltage phase preceding the rising and falling edges, no charge signal was detected, indicating that both v_0_ and v are approximately zero. This implies that the resultant force acting on the charge is negligible, meaning the electric force balances the material stress, and the charge remains stationary. Conversely, charge signals were detected during the rising and falling edges, confirming that the charge undergoes vibration.

### 2.3. Influence Chain: Influencing Factors Change—Forces Application on Charges—Charges Dynamic Behaviors—Electrical Tree Formation and Silicone Gel Insulation Degradation

#### 2.3.1. From Influencing Factors Change to Forces Application on Charges

The pulse voltage slew rate is reflected by the extremely short pulse edges. An increase in pulse voltage slew rate causes rapid variations in the pulse electric field, leading to carrier migration lagging behind electric field changes. This results in instantaneous intensification of the interfacial electric field, lowering the injection barrier and substantially boosting the carrier injection rate. Simultaneously, charges stimulated by the rapid electric field changes readily overcome trap energy level barriers, shortening the time scales for trapping and detrapping. This creates a rapid trapping–detrapping dynamic cycle. Therefore, an increased pulse voltage slew rate induces extremely violent charge dynamics and triggers intense energy release. By observing the experimental data in Section 4.1, it can be seen that as the pulse voltage slew rate increases, the amplitude of charge vibrations significantly grows. This indicates that charges possess greater velocity, the change in kinetic energy increases, and simultaneously, the time-varying value decreases. Consequently, according to the formula, the resultant force can be calculated to increase with the rise in pulse slew rate.

The effects of the superimposed DC amplitude and duty cycle are exhibited during the prolonged constant-voltage phase of the pulse. As both voltage amplitude and duty cycle increase, field emission and thermal emission intensify, boosting carrier injection into the electrodes. Simultaneously, carrier trapping efficiency improves, leading to sustained static accumulation of space charge and elevated charge density. The additional electric field generated by space charge accumulation superimposes with the applied external electric field, forming a locally distorted electric field. This increases the electric field strength, intensifies the electric force acting on molecules, and alters their state under force. As shown by the experimental data in Section 4.1: with increasing superimposed DC amplitude and duty cycle, the amplitude of charge vibration increases. Combining the relationship between charge velocity and voltage amplitude with the formula for the theorem of kinetic energy, this similarly indicates that the resultant force increases accordingly.

#### 2.3.2. From Forces Application on Charges to Charges Dynamic Behaviors

Changes in the force state of space charge will significantly alter the intensity of charge vibration through two pathways: direct collision and distortion-driven vibration. On the one hand, accelerated space charge undergoes direct collisions with insulating molecules during migration, driving charge vibration. This behavior may also trigger chain collisions, further expanding the spatial extent of charge vibration. On the other hand, the additional electric field generated by space charge accumulation combines with the applied electric field to form a localized distorted electric field. This distorted field also drives charge vibrations. Consequently, changes in the force state of space charge cause it to be driven by both the electric field force and the distorted electric field, resulting in increased vibration amplitude and heightened vibration intensity. These charge vibrations reflect both the dynamic changes and static accumulation of the charge.

The positive feedback coupling mechanism between charge motion and localized electric field enhancement forms a continuous, self-amplifying closed loop: the directional migration, interfacial accumulation and reciprocating vibration of charges triggered by the steep edges of the pulse directly cause non-uniform distribution of space charge in silicone gel, which in turn induces distortion and enhancement of the localized electric field; the enhanced local electric field further reduces the charge injection barrier and increases the electrical force acting on the charges, in turn aggravating the transport lag, trapping–detrapping cycle and vibration behavior of charges, and intensifying the non-uniform distribution of space charge. This closed loop continuously amplifies the intensity of charge motion and localized electric field enhancement, and the increase in superimposed DC amplitude and duty cycle raises the baseline level of space charge accumulation and local field distortion, which further accelerates the self-reinforcing process of the loop.

#### 2.3.3. From Charges Dynamic Behaviors to Electrical Tree Formation and Silicone Gel Insulation Degradation

The dynamic behaviors of charge injection, extraction, trapping, detrapping, and recombination intensify. The energy released during these processes causes microscopic chemical bond breakage through mechanisms such as high-energy electron generation, light radiation, and induced charge dissociation. The cumulative effect of chemical bond disruption triggers molecular chain scission and reduced crosslinking density. Microcracks form within the material, and microstructural damage begins to manifest. Simultaneously, localized electric field distortion intensifies, disrupting the initial uniform electric field distribution within the insulator. The electric field strength significantly increases in charge accumulation zones and at insulator defects. Under the influence of strong electric fields at material defects, residual gases within the defects ionize, forming flow channels. Active substances generated during discharge react oxidatively with insulating molecules, causing molecular chain embrittlement and scission. High-energy particles continuously collide with insulating molecules, further breaking molecular chains and propagating microcracks outward. This forms distinct discharge channels that expand into electrical trees, ultimately penetrating the electrodes to cause breakdown and insulation failure [30]. In summary, intensified charge dynamics accelerate structural damage within the insulation, exacerbate electric field distortion, and trigger and amplify partial discharges. Through the combined effects of electrothermal coupling, the formation of electrical trees and the degradation of insulation in silicone gel encapsulation materials are promoted.

#### 2.3.4. Fundamental Mechanistic Differences Between Pulse-Induced Charge Vibration and Conventional AC/DC Electrical Stresses

Pulse-induced charge vibration differs fundamentally from conventional AC and pure DC stress mechanisms in its physical origin, action mode, and microscopic degradation pathway. Unlike steady-state unidirectional charge accumulation under DC or periodic long-range charge transport under AC, this vibration is a transient, impulsive electro-mechanical behavior occurring exclusively at steep pulse edges. It is driven by the force imbalance of trapped charges when the pulse edge time is shorter than the charge relaxation time in silicone gel. Instead of relying on impact ionization or long-term electric field distortion, it causes direct molecular chain scission via localized reciprocating charge vibration. The pulse voltage slew rate, rather than the absolute voltage amplitude, acts as the dominant factor governing this degradation behavior. This unique mechanism explains the premature silicone gel insulation failure in wide-bandgap power devices that cannot be predicted by traditional AC/DC aging models.

### 2.4. Comparative Analysis of the Influence Mechanisms of Pulse Voltage Slew Rate, Superimposed DC Amplitude, and Duty Cycle

**Figure 13:**
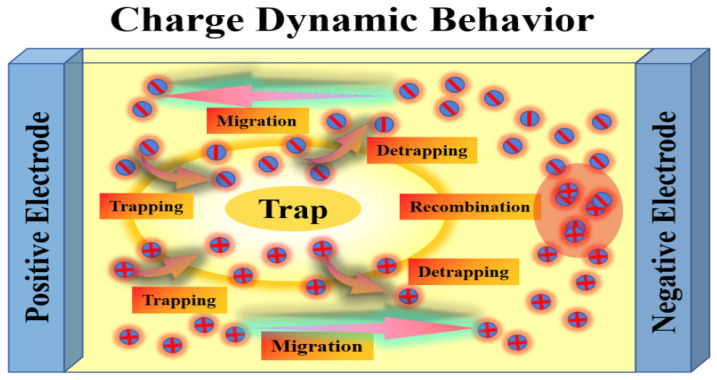
Schematic diagram of space charge dynamic behaviors.

As shown in Figure 13, the increased slew rate of pulse voltage intensifies the dynamic behaviors of charge, including trapping, detrapping, migration, and recombination. The high-frequency, rapid vibration of charges over extremely short durations is accompanied by intense energy release. High-energy carriers collide with molecules at high speeds, directly disrupting chemical bonds and causing their breakage. This generates microcracks, leading to microstructural damage. Simultaneously, high-energy electrons generated by partial discharges further bombard molecules, accelerating chemical bond rupture. Heat released during discharge promotes microcrack propagation, forming discharge channels that evolve into electrical trees. Ultimately, these structures penetrate the electrode, triggering breakdown.

**Figure 14:**
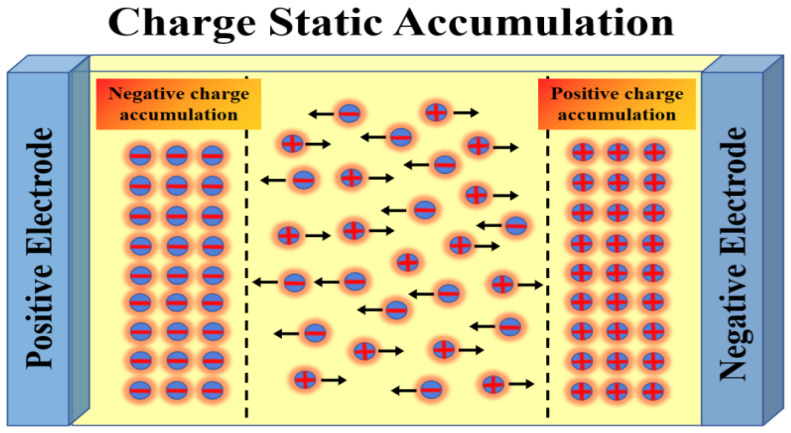
Schematic diagram of space charge static accumulation.

As shown in Figure 14, the superimposed DC amplitude and duty cycle increase, reflected in elevated charge density—that is, static charge accumulation. The additional electric field generated by spatial charge accumulation superimposes with the applied external electric field, forming a locally distorted electric field, which is consistent with the conclusion—the increasing number of charges accumulated near the electrodes distorts the electric field and eventually leads to breakdown by Zhang, S.Y. et al. [31]. This distortion elevates the electric field strength, causing the electric force to continuously stretch chemical bonds. This stretching induces bond fatigue and relaxation, leading to a gradual decay in bond energy. Under the prolonged influence of static charge accumulation, damage to chemical bonds slowly accumulates and evolves, gradually generating microscopic defects that propagate. Ultimately, this process results in insulation degradation.

**Figure 15:**
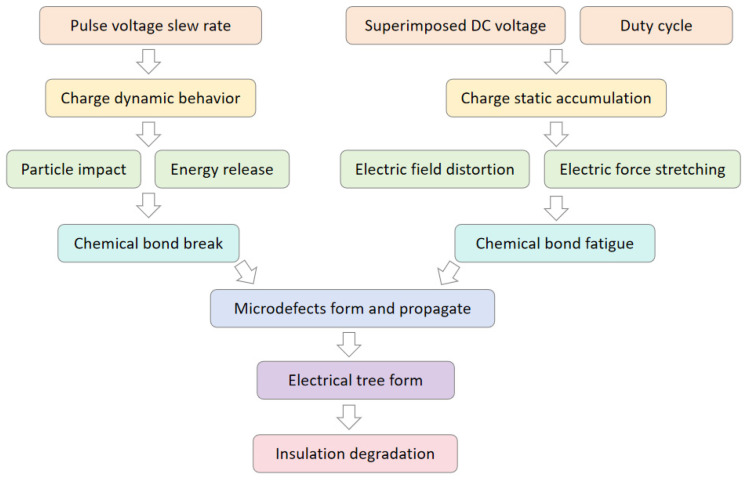
Comparative analysis of the influence mechanisms of pulse voltage slew rate, superimposed DC amplitude, and duty cycle.

The dynamic behaviors of electric charges are extremely intense, accompanied by instantaneous high-density energy release. This can trigger chain reactions, with the released energy directly exceeding the load-bearing threshold of the material’s microstructure. This forms irreversible discharge channels that rapidly propagate, causing insulation degradation within a short timeframe [32]. In contrast, the static accumulation of space charge persists over extended periods and progresses relatively slowly, driven solely by the gradual action of electric field forces. Material damage can partially recover when the electric field weakens, and insulation degradation only occurs after prolonged exposure. Therefore, compared to the violent dynamic behaviors of charge, the static accumulation of charge is more moderate and gradual. Figure 15 presents a comparative analysis of the influence mechanisms of pulse voltage slew rate, superimposed DC amplitude, and duty cycle.

Combining experimental data from previous space charge measurements: when the pulse voltage slew rate increases, the variation in charge vibration amplitude exceeds 50 times (from 0.02324 V to 1.27614 V). In contrast, when the superimposed DC amplitude and duty cycle increase, the variation in charge vibration amplitude is approximately 172% and 16.5%, respectively. The pulse voltage slew rate exerts a significantly greater influence on charge vibration than the superimposed DC amplitude and duty cycle. The aforementioned theoretical analysis of charge dynamic variation and static accumulation is further substantiated. Consequently, it is concluded that the influence of pulse voltage slew rate surpasses that of superimposed DC amplitude and duty cycle. Correspondingly, the impact of charge dynamic behaviors outweighs that of charge static accumulation.

### 2.5. Practical Engineering Implications

The findings provide direct theoretical guidance and quantitative reference for the insulation design and reliability optimization of the silicone gel packaging of high-voltage power devices and equipment, providing a feasible engineering solution for reducing the cost of device maintenance and replacement caused by silicone gel insulation degradation.

For those devices and equipment operating under DC superimposed pulse conditions with steep edges, this work highlights that suppressing excessive pulse voltage slew rate should be prioritized as the primary measure to mitigate silicone gel insulation degradation, rather than only focusing on the regulation of static DC amplitude and duty cycle.

For the actual packaging structure, the proposed strategy of suppressing pulse voltage slew rate can be integrated into the packaging design process: for example, optimizing the layout of snubber circuits in the device packaging to reduce the steepness of pulse edges at the chip terminals, and adjusting the thickness and filling process of silicone gel in key areas around bonding wires to alleviate charge static accumulation and dynamic behaviors.

Furthermore, the experimental data and microscopic degradation mechanism revealed in this research can also provide a scientific basis for the formulation of industry test specifications for silicone gel insulation serving in complex DC superimposed pulse conditions, filling the gap in the assessment of nanosecond-level pulse slew rate in existing insulation evaluation systems.

## 3. Conclusions

This experiment investigated and verified the primary factors and mechanisms influencing the insulation degradation of silicone gel materials through electrical tree and space charge vibration experiments. The main conclusions are as follows:

(1) Using the controlled variables method, the effects of pulse variation value, superimposed DC amplitude, and duty cycle on the degradation of silicone gel insulation were quantified and compared. The results indicate that, with the pulse rise time held constant, pulse variation value has the greatest impact on silicone gel insulation degradation, followed by superimposed DC amplitude, while duty cycle has the least effect. The increase in superimposed DC amplitude and duty cycle are reflected as an increase in static charge accumulation.
(2) It is known that when the pulse rise time remains constant, the pulse variation value has the greatest impact on silicone gel insulation degradation. Furthermore, the pulse rise time exerts a greater influence on silicone gel insulation degradation than the pulse variation value. The pulse voltage slew rate is precisely composed of both the pulse variation value and the rise time. Therefore, the most critical factor affecting silicone gel insulation degradation is the pulse voltage slew rate. An increase in the pulse voltage slew rate is reflected as intensified dynamic behaviors of charge at the pulse edge.
(3) Increased voltage slew rate, superimposed DC amplitude, and duty cycle alter the force state of charges, thereby modifying charge accumulation and dynamic behaviors. This ultimately influences the formation of electrical tree and the insulating degradation of the silicone gel materials. Within this influence chain, the voltage slew rate reflects the intense dynamic behaviors of charges, while the superimposed DC amplitude and duty cycle reflect the gradual static accumulation of charges. The impact of dynamic charge behaviors is greater than that of static charge accumulation. The microscopic mechanism of silicone gel material insulation failure is reasonably explained through this influence chain, and the pulse voltage slew rate is further identified as the most critical influencing factor.

## 4. Materials and Methods

### 4.1. Electrical Tree Experiment

#### 4.1.1. Sample Selection and Preparation

**Figure 16:**
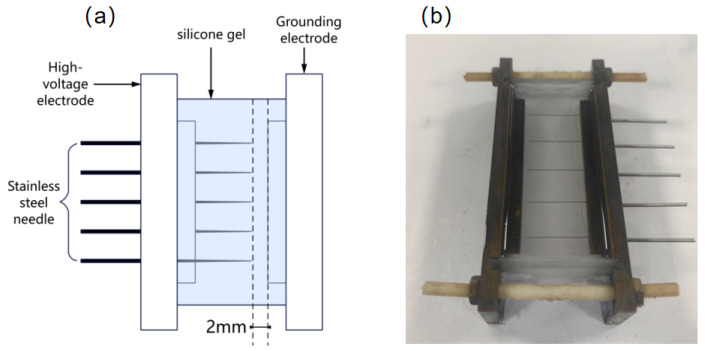
Silicone gel electrical tree mold. (**a**) Schematic diagram and (**b**) physical diagram.

The silicone gel used in this experiment is Wacker-612. Components A and B of the silicone gel were mixed in a 1:1 mass ratio, then placed in a centrifuge for thorough mixing for 120 s. The mixture was subsequently poured into a mold (70 mm × 38 mm × 14.8 mm) with a needle plate spacing of 2 mm. The mold was then placed in a constant-pressure chamber for vacuum processing to remove gases from the mixture and prevent bubbles from affecting the experimental results. After vacuuming until no further bubbles formed in the mold, the sample was removed. Finally, the sample was placed in a vacuum oven and cured at 110 °C for 1 h, yielding the highly tough, transparent silicone gel elastomer shown in Figure 16.

#### 4.1.2. Experimental Platform

**Figure 17:**
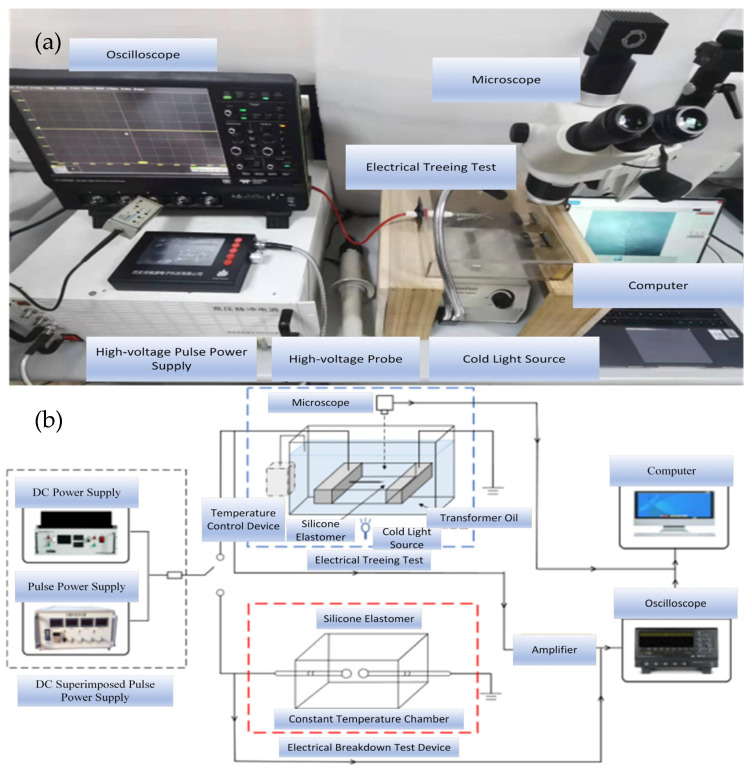
Electrical tree experiment platform. (**a**) Schematic diagram and (**b**) physical diagram.

The electrical tree experimental platform, as shown in Figure 17, consists of a high-voltage DC power supply, a square wave pulse power supply, a coupled superposition circuit, an electrical tree mold, a remote-controlled sliding rail, a halogen lamp, a microscope, a temperature control system, and a computer. The high-voltage DC power supply, manufactured by Dongwen High-Voltage Power Supply Company (Tianjin, China), can output a DC voltage with an adjustable amplitude of 0–10 kV and negative polarity. The square wave pulse power supply utilizes the HVP-10B model manufactured by Xi’an Lingfengyuan Electronic Technology Co., Ltd. (Xi’an, China). It outputs a negative polarity square wave pulse voltage with adjustable amplitude (0–10 kV), duty cycle (0–50%), frequency (0–10 kHz), and edge time (50 ns).

To achieve interference-free superposition between high-voltage DC signals and square-wave pulses/AC signals while protecting the equipment. This experiment employs a coupling superposition circuit composed of diodes, capacitors, and resistors, whose structure is shown in Figure 17. Due to the unidirectional conduction characteristic of diodes, the negative polarity voltage generated by the DC power supply can pass through the diode to the test specimen, while the voltage signal generated by the square wave pulse/AC power supply is blocked by the diode, preventing damage to the DC power supply. The capacitor’s ability to block DC signals while conducting AC signals allows the alternating signal from the square wave pulse/AC power source to pass through. However, the DC signal from the high-voltage DC power source is blocked by the capacitor, preventing the power supply from being damaged by the high-voltage DC signal. The resistor serves a protective function: if the electrical tree mold breaks down, the resistor limits the current flowing through the circuit.

By altering the high-voltage non-inductive resistor R1 and the capacitance Cx of the test specimen, the time constant τ of the filter circuit’s charging and discharging is formed, enabling different pulse voltage rise times. The expression for the time constant is: τ = R1 × Cx. Therefore, the time constant τ can also be adjusted by modifying the value of resistor R1, thereby generating pulses with varying rise and fall times. In the experiment, the high-voltage non-inductive resistor R1 was set to values of 250 Ω, 2500 Ω, 10 kΩ, and 300 kΩ, yielding rise/fall times of 100 ns, 1 μs, 10 μs, and 100 μs, respectively.

The microscope used is the Motic SMZ-171TL optical microscope (Motic, Xiamen, China), with adjustable magnification ranging from 0× to 50×. During testing, the microscope is positioned above the needle tip. The image signal is transmitted to a computer via a Charge-Coupled Device (CCD) camera, enabling observation and recording of the electrical tree characteristics at the needle tip. To ensure uniform brightness within the field of view, a halogen cold light source illuminates the sample, providing supplementary lighting while preventing temperature increases caused by light source overheating. The dendrite mold is mounted on a remote-controlled sliding rail. Using a remote controller, the rail position can be adjusted to sequentially expose the five needles within the microscope’s field of view, enabling observation of dendrite characteristics at different needle tips.

#### 4.1.3. Selection of Quantitative Parameters

**Figure 18:**
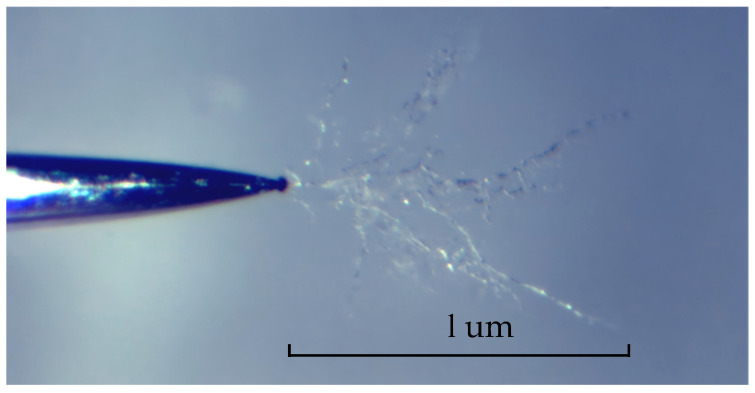
Electrical tree image under microscope.

A negative square wave pulse voltage with a frequency of 500 Hz and variable pulse absolute value, variation value, rise time, and duty cycle was applied to the sample. To quantify the extent of each influencing factor on the degradation of silicone gel insulation, the following parameters were selected: length of electrical tree, growth rate, number of electrical tree initiation, electrical tree initiation time, number of electrical tree that broke down, and electrical tree breakdown time, all measured within the 1 h voltage application period. Figure 18 shows an optical microscope image of the electrical tree. The calculation of each parameter is as follows.

(1) Length l (um): The final length of the electrical tree along the needle direction when a voltage is applied for 1 h or immediately before breakdown occurs.
(2) Growth rate v (um/min): The start time for the electrical tree growth is recorded as (min). If no breakdown occurs, the end time is set to 60 min, and the electrical tree growth time is calculated as: if breakdown occurs, the end time is set to the breakdown time (min), and the electrical tree growth time is calculated as. From this, the electrical tree growth rate can be determined as.
(3) Number of electrical tree initiation: The number of pins exhibiting treeing phenomena (including breakdown) within one hour of voltage application.
(4) Initiation time (min): The time from the instant the voltage is applied until each needle exhibits treeing.
(5) Number of electrical tree that broken down: The number of pins that broke down within one hour of voltage application.
(6) Breakdown time (min): The time interval from the instant voltage is applied until breakdown occurs in each needle.

For the aforementioned quantitative parameters, longer electrical tree length, faster growth rate, greater number of electrical tree initiation, shorter initiation time, higher number of electrical tree that broke down, and shorter breakdown time all indicate more severe insulation degradation in the silicone gel samples.

#### 4.1.4. Experiment Preparation

Using the electrical tree experimental platform, the first part of the electrical tree experiment examines the three primary characteristics of applied pulse voltage—pulse variation value, superimposed DC amplitude, and duty cycle—to investigate their respective impacts on the degradation of silicone gel insulation. Prior to experimentation, an appropriate pulse voltage must be identified as the baseline voltage. Samples exhibited rapid breakdown under pulse voltage from 0 to −10,000 V, which hindered experimental observation. A pulse voltage ranging from 0 to −5000 V was applied, no electrical tree occurred at this level, whereas electrical tree appeared under the pulse voltage from 0 to −6000 V. This voltage allowed sufficient time for electrical tree development without causing premature breakdown. Therefore, the pulse voltage from 0 to −6000 V was selected as the experimental reference voltage.

This experiment employs a variable-controlling approach, gradually increasing the voltage amplitude in steps. Using a pulse voltage source and a DC voltage source, three sets of DC-superimposed pulse voltages were generated. A negative-polarity square wave pulse voltage with a frequency of 500 Hz and a fixed rise time of 100 ns was applied to the sample.

The first group maintains constant pulse variation and duty cycle while varying only the superimposed DC amplitude to investigate its effect on sample insulation degradation. Based on the experimental reference voltage ranging from 0 to −6000 V pulse voltage, DC voltages of +3000 V, −3000 V, and −6000 V were superimposed respectively. This yielded pulse voltage sets ranging from 3000 V to −3000 V, 0 to −6000 V, −3000 V to −9000 V, and −6000 V to −12,000 V. Each set of pulse voltages was applied to four samples, with the experiment repeated five times.

The second group maintained constant superimposed DC amplitude and duty cycle, altering only the pulse variation value to investigate its impact on sample insulation degradation: two sets of pulse voltages ranging from 0 to −3000 V and from 0 to −9000 V were applied to the samples, with each experiment repeated five times. Combined with the previous group’s experiments featuring pulse voltages from +3000 V to −3000 V and from −3000 V to −9000 V. Experimental phenomena under the 0 to −3000 V and +3000 V to −3000 V pulse voltage sets were compared, as were those under the −3000 V to −9000 V and 0 to −9000 V sets.

The third group maintains constant pulse variation and superimposed DC amplitude while varying only the duty cycle to investigate its effect on sample insulation degradation. Maintaining the pulse voltage constant at 0 to −6000 V, with a pulse voltage period of 2 us at a pulse voltage frequency of 500 Hz, the duration of the applied negative polarity voltage varied between 0.2 us, 0.6 us, and 1 us, yielding duty cycles of 10%, 30%, and 50%. This produced three sets of pulse voltages with different duty cycles, each applied to three samples. The experiment was repeated five times.

Observe and record the length, growth rate, number of electrical tree initiation, initiation time, number of electrical tree that broken down, and breakdown time of electrical tree growth under different superimposed DC pulse voltages within 60 min. Remove outliers and calculate the average values. Compare and analyze the impact of pulse variation, superimposed DC amplitude, and duty cycle on sample insulation degradation. Longer electrical tree length, faster growth rate, greater number of electrical tree initiation, shorter initiation time, higher number of electrical tree that broken down, and shorter breakdown time indicate that the respective factor exerts a more destructive effect on the insulation degradation of the silicone gel sample, signifying a greater impact.

The second part of the electrical tree experiment is to investigate the effect of pulse rise time on the insulation degradation of silicone gel samples. Four sets of negative square wave pulse voltages were applied to the samples. These pulses had a frequency of 500 Hz, a duty cycle of 50%, and a voltage range of 0 to −6000 V. The rise times were set to 100 ns, 1μs, 10μs, and 100μs respectively, so their rising edge slopes are $-6\times 10^{7}\;kV/s$, $-6\times 10^{6}\;kV/s$, $-6\times 10^{5}\;kV/s$ and $-6\times 10^{4}\;kV/s$ respectively. Apply this set of pulse voltages to the samples respectively, and record the following parameters over a 60 min period for different rise times: length of electrical tree, growth rate of electrical tree, number of electrical tree initiation, electrical tree initiation time, number of electrical tree that broke down, and electrical tree breakdown time. Remove outliers and calculate the average values. Conduct a comprehensive analysis of the impact of rise time on the insulation degradation of the silicone gel samples.

### 4.2. Charge Vibration Experiment

#### 4.2.1. Sample Selection and Preparation

This experiment utilizes Wacker-612 silicone gel. A small amount of silicone gel components A and B were taken and mixed in a 1:1 mass ratio. The mixture was then placed in a centrifuge for thorough mixing over 120 s. Subsequently, it was placed in a constant-pressure chamber for vacuum processing to remove gases from the mixture and prevent bubbles from affecting the experimental results. After vacuuming until no further bubbles formed in the mold, the sample was removed. Finally, the sample was placed in a vacuum oven and cured at 110 °C for 1 h, yielding a highly resilient, transparent silicone gel elastomer.

#### 4.2.2. Experimental Platform

**Figure 19:**
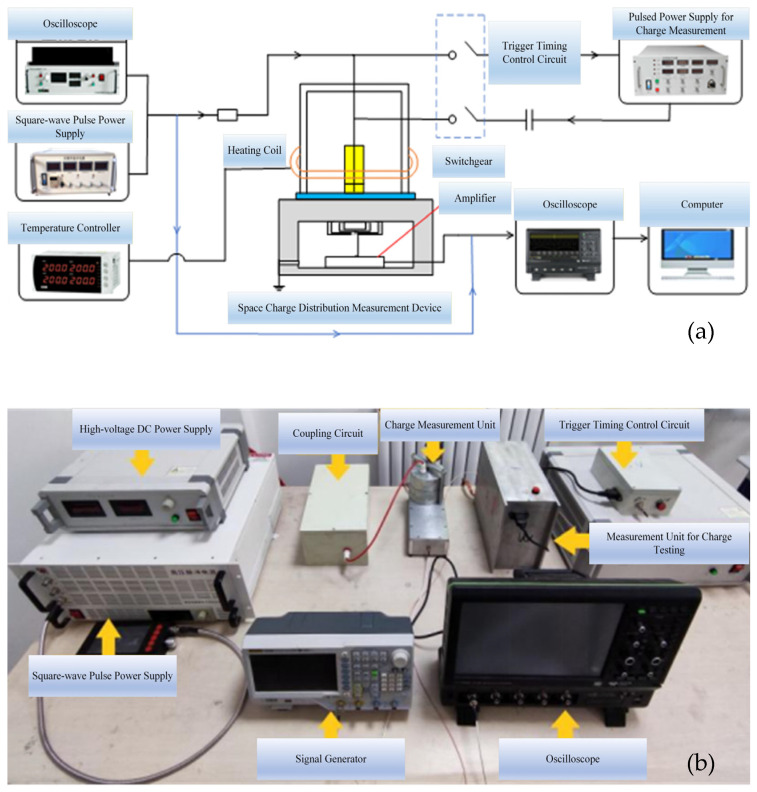
Charge vibration experiment platform. (**a**) Schematic diagram and (**b**) physical diagram.

A space charge vibration experimental platform was established. The overall schematic diagram of the testing system is shown in Figure 19a. This system primarily consists of a high-voltage square-wave pulse power supply, a DC power supply, a coupling circuit, a space charge measurement unit, a PEA test unit, a trigger control circuit, a LeCroy oscilloscope, and a computer. The silicone gel elastomer is removed and spread into a thin layer, positioned between the high-voltage electrode and the ground electrode. A polarization voltage is applied to the silicone gel sample through the coordinated operation of the pulse power supply and the high-voltage DC power supply, causing space charge to generate and accumulate within the sample. The assembled experimental platform is shown in Figure 19b.

Based on the principle of measuring polarized space charge using piezoelectric sensing, considering that the abrupt change in voltage amplitude at the pulse edge may induce specific charge motion behavior accompanied by acoustic signals and other responses, it is feasible to detect the unique electroacoustic response generated by space charge under pulse voltage excitation using a PVDF (Polyvinylidene Fluoride) piezoelectric sensor.

**Figure 20:**
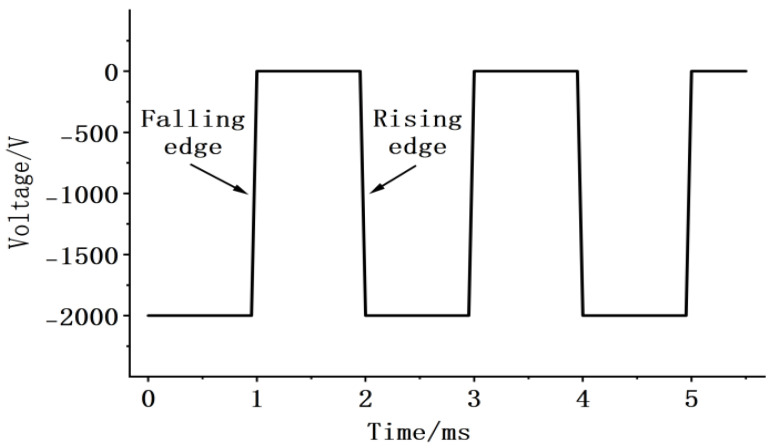
Negative polarity pulse voltage diagram.

**Figure 21:**
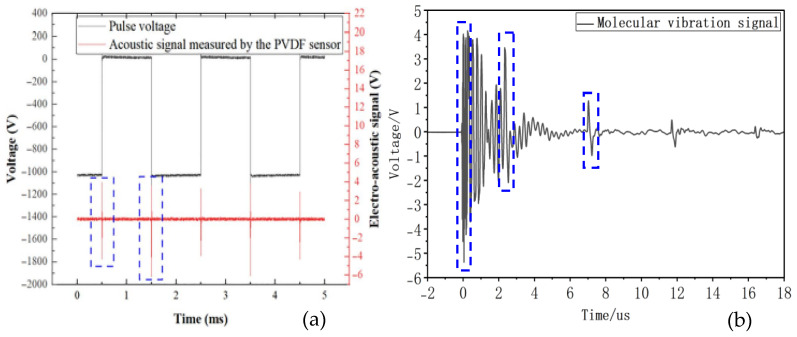
Charge vibration waveform diagram. (**a**) Electro-acoustic signal at the rising and falling edges. (**b**) Amplified charge vibration waveform at the rising edge.

A negative-polarity pulse voltage with an amplitude of 2000 V and a frequency of 500 Hz is applied to the sample. By varying the rise and fall slope rates and duty cycle of the pulse voltage, the charge characteristics at the nanosecond-level pulse voltage edges are detected using a PVDF piezoelectric sensor. The negative-polarity pulse voltage waveform is shown in Figure 20. In this study, the “rising edge” refers to the process of increasing voltage amplitude, while the “falling edge” denotes the process of decreasing voltage amplitude. For instance, in a negative-polarity pulse voltage, the rising edge corresponds to the voltage decreasing from 0 to −*n* kV, and the falling edge corresponds to the voltage increasing from −*n* kV back to 0. A trigger control circuit precisely locates the signal detected by the PVDF piezoelectric sensor at the pulse rising edge, enhancing test resolution and enabling accurate waveform identification and analysis. The identified and amplified waveform is shown in Figure 21, which can be clearly divided into three distinct sections (highlighted by the blue boxes in Figure 21b).

**Figure 22:**
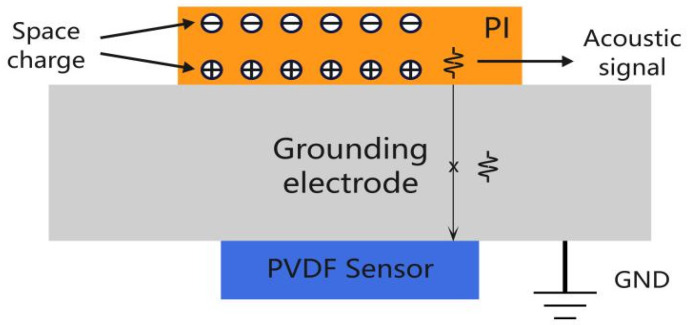
Schematic diagram of sound wave signal propagation.

The first component may be an electromagnetic signal generated by the sudden voltage change at the pulse edge. Since electromagnetic waves propagate at extremely high speeds—approximately the speed of light—they are captured by the sensor the instant they are generated. The second waveform component may be an ultrasonic signal produced by internal charges within the material. Due to the presence of a grounded aluminum electrode plate between the sample and the sensor, the signal generated by the charge experiences a certain delay before being received by the sensor. The schematic diagram of its propagation process is shown in Figure 22, and the delay calculation formula is as follows:

$$T_{daley}=\frac{x}{v}$$

In the above equation, *x* represents the thickness of the aluminum electrode plate, and *v* denotes the speed of sound. In this system, the aluminum electrode has a thickness of 15 mm, and the speed of sound in aluminum is approximately 6300 m/s. Calculating the delay yields approximately 2.4 μs, which aligns closely with the experimentally measured result. Additionally, the third waveform segment arises from the signal undergoing refraction and reflection within the aluminum electrode plate, traversing a path twice the thickness of the plate. Consequently, its delay should be 4.8 μs. This value aligns with the experimentally measured delay between the second and third waveforms. Moreover, the third waveform exhibits a noticeable attenuation in amplitude compared to the second waveform after refraction and reflection. This confirms that the signal detected by the piezoelectric sensor originates from acoustic waves generated by the vibration of internal charges within the material, stimulated by the pulse edge.

#### 4.2.3. Selection of Quantitative Parameters

**Figure 23:**
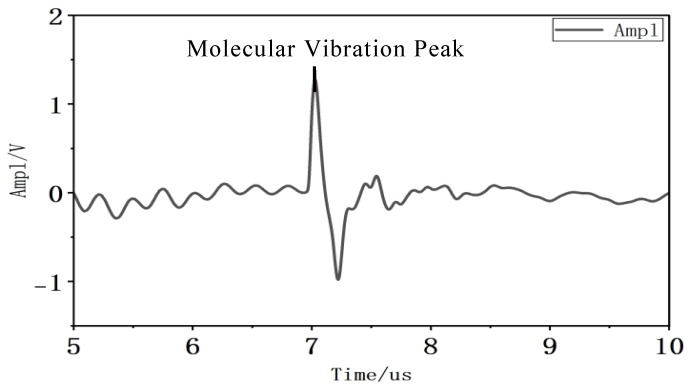
Schematic diagram of charge vibration peak value.

Under a pulse electric field, bound charges experience forces that induce vibration. Beyond bound charges, additional charges exhibit dynamic behaviors such as trapping, detrapping, migration, and recombination—phenomena difficult to measure directly through testing [33]. However, since both types of charges exhibit identical variation patterns under the influence of forces in the pulse electric field, the intensity of charge dynamics can be indirectly reflected through charge vibration phenomena. Based on this analysis, the third portion of the waveform (around 7.2 us), which is less disturbed and easier to observe, was selected for peak analysis. The vibration peak of this portion was identified and adopted as a parameter quantifying the intensity of charge vibration, thereby reflecting charge dynamic behaviors through this intensity. A schematic diagram of the charge vibration peak is shown in Figure 23. A larger vibration peak indicates more intense charge vibration and more vigorous charge dynamic behaviors.

#### 4.2.4. Experimental Preparation

Apply a negative-polarity pulse voltage ranging from 0 to −2000 V with a pulse frequency of 500 Hz and a rise time of 100 ns. Superimpose a DC voltage of −2000 V onto this pulse voltage, resulting in a negative-polarity pulse voltage ranging from −2000 V to −4000 V with a pulse frequency of 500 Hz and a rise time of 100 ns. Apply six sets of pulse voltages with varying pulse amplitudes (−2000 V, −4000 V) and duty cycles (10%, 30%, 50%) to the sample. Test each set five times. Observe charge vibration waveforms and record vibration amplitudes to compare the intensity of charge vibrations under different amplitude and duty cycle conditions. Finally, apply pulse voltages to the sample with a frequency of 500 Hz, a duty cycle of 50%, and pulse voltages ranging from 0 to −2000 V. The rise times are 100 ns, 1 us, 10 us, and 100 us. Repeat the experiment five times. Observe the charge vibration waveforms and record the vibration amplitudes. The magnitude of the influence of the pulse variation rate was compared with that of the superimposed DC amplitude and duty cycle. The charge dynamic behaviors were reflected through the charge vibration amplitude.

## Data Availability

The original findings presented in this work are fully included in the article. Further details may be obtained by contacting the corresponding author.
